# Biasogram: Visualization of Confounding Technical Bias in Gene Expression Data

**DOI:** 10.1371/journal.pone.0061872

**Published:** 2013-04-16

**Authors:** Marcin Krzystanek, Zoltan Szallasi, Aron C. Eklund

**Affiliations:** 1 Center for Biological Sequence Analysis, Department of Systems Biology, Technical University of Denmark, Lyngby, Denmark; 2 Children's Hospital Informatics Program at the Harvard-MIT Division of Health Sciences and Technology (CHIP@HST), Harvard Medical School, Boston, Massachusetts, United States of America; Cleveland Clinic Lerner Research Institute, United States of America

## Abstract

Gene expression profiles of clinical cohorts can be used to identify genes that are correlated with a clinical variable of interest such as patient outcome or response to a particular drug. However, expression measurements are susceptible to technical bias caused by variation in extraneous factors such as RNA quality and array hybridization conditions. If such technical bias is correlated with the clinical variable of interest, the likelihood of identifying false positive genes is increased. Here we describe a method to visualize an expression matrix as a projection of all genes onto a plane defined by a clinical variable and a technical nuisance variable. The resulting plot indicates the extent to which each gene is correlated with the clinical variable or the technical variable. We demonstrate this method by applying it to three clinical trial microarray data sets, one of which identified genes that may have been driven by a confounding technical variable. This approach can be used as a quality control step to identify data sets that are likely to yield false positive results.

## Introduction

Discovery of novel biomarkers is an important goal in many areas of biomedical research. In particular, we are interested in predictive tumor biomarkers that can aid in patient treatment decisions by identifying the anti-cancer therapies that are most likely to cure a given patient. One approach to biomarker discovery is to obtain gene expression profiles of pre-treatment tumor specimens from a cohort of patients, and to search for genes or combinations of genes whose expression is correlated with individual drug response.

There are at least two potential hurdles to associative, or data-driven, approaches to biomarker discovery. First, multiple hypothesis testing is likely to generate false positives if not considered in the analysis; fortunately, this problem is fairly well-understood in principle [1]. Second, the consistent measurement of gene expression is particularly tricky in large clinical trials, due to extraneous technical factors, such as batch effects and varying specimen quality, that cause non-random measurement error and lead to large sets of genes with spurious correlation to the technical factor [2], [3].

A particularly undesirable situation can arise if extraneous technical factors are correlated, even weakly, with the clinical outcome of interest in a particular data set. In this case, the (potentially large number of) genes affected by the technical factor have an increased likelihood of appearing to be correlated with clinical outcome, and standard methods for controlling false positives are inadequate. Here we propose a simple visualization method to assess the impact of extraneous technical factors on false positives in associative gene expression studies.

## Methods

The input data consists of three elements: 1) An gene expression matrix **X** with dimension *n*×*m*, where *n* is the number of probes (or probe sets) and *m* is the number of specimens, and the matrix element *x_ij_* represents the normalized expression level of probe *i* in specimen *j*. 2) An outcome vector *Y* of length *m*, where element *y_j_* indicates the outcome (or other variable of interest) of specimen *j*. In practice, *Y* might be coded as a binary response (e.g. 1, 0 for resistant vs. sensitive), as coded levels (e.g. 1–5 for the Miller-Payne score), or as a continuous variable (e.g. change in tumor volume). 3) A bias vector *B* of length *m*, where each element *b_j_* indicates the relative influence of technical bias (or other nuisance variable) on the data from specimen *j*. The vectors *Y* and *B*, as well as each gene vector *x_i•_*, are individually scaled to have a mean of zero and unity Euclidean length.

The first step is to identify a quantifiable source of technical bias that is expected to affect gene expression measurements but is not caused by cellular or physiological factors. Several such technical factors affect the biological specimen, e.g. RNA yield and integrity can be quantified by chromatography [4]. Batch identifiers may indicate technical bias; but in the biasogram this is only appropriate if there are exactly two batches (although >2 batches can be analyzed using multiple biasograms; see Case Study 3). Other factors may be available from microarray processing software, such as a quality score or signal-to-noise ratio. In some cases the normalization algorithm itself may be a source of bias [5]. Several technical factors can be inferred post hoc from raw microarray data; e.g. the level of negative control probes can indicate changes in the noise floor, or the width of the distribution of expression values can indicate dynamic range [3]. We do not necessarily expect the bias metrics to be independent of each other. Furthermore, we recognize that some of these bias metrics are likely to reflect multiple sources of technical bias; it is difficult to separate these effects due to normalization at various steps of the microarray protocol.

**Figure 1:**
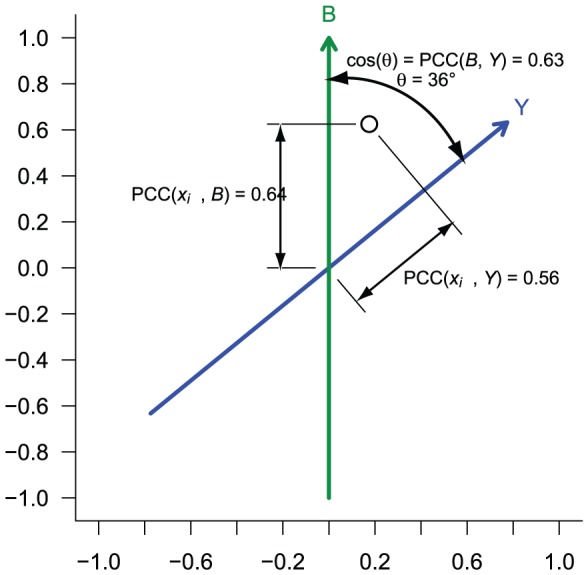
Interpretation of the “biasogram”. The biasogram is an orthogonal projection of a gene expression matrix onto a plane defined by previously defined vectors quantifying an outcome *Y* and bias *B* for each patient. Each point represents a gene, or probe set. *Y* and *B* define axes for a skew coordinate system in which the position of each point indicates its Pearson correlation coefficient with *Y* and *B*, respectively. The angle between *Y* and *B* represents the correlation between *Y* and *B*. For the sake of explanation, only a single gene is represented in this figure. In general, a biasogram for many thousands of genes can be represented by a heatmap rather than by individual points. PCC, Pearson correlation coefficient.

The second step is to identify an appropriate orthogonal projection. The gene vectors *x_i•_*, as well as the vectors *Y* and *B*, can be interpreted as points in *m*-dimensional space **R** ***^m^***, with each dimension corresponding to an individual experiment. The orthogonal projection matrix **P** is chosen such that all points in **R** ***^m^*** are projected into a two-dimensional subspace *S* of **R** ***^m^*** that includes *Y* and *B*. Thus, the projection “flattens” the genes onto the most interesting plane – the one containing the two variables of interest, *Y* and *B*. Conveniently, this projection of the data has an intuitive geometric interpretation: *Y* and *B* define the axes in a skew coordinate system, where the cosine of the angle between the axes is equal to the Pearson correlation coefficient (PCC) between *Y* and *B*. Furthermore, the position of a gene vector *x_i•_* along these axes indicates the PCC between each gene and *Y* or *B* (**Fig. 1**).

In practice, we calculate **P** by applying singular value decomposition (SVD) to a 2×*m* input matrix created by joining the scaled *Y* and *B* vectors. The resulting V* matrix (whose columns contain the right singular vectors of the 2×*m* input matrix) is a projection matrix that meets our criteria by bringing *Y* and *B* entirely into two dimensions. However **P** *Y* and **P** *B* both have components in each of the two dimensions; in order to provide a more intuitive reference point, we give **P** an additional “rotation”such that **P** *B* falls entirely in the second dimension (i.e. **P** *B* aligns with the positive vertical axis), and **P** *Y* has a positive value in the first dimension (defining the skew, but generally more horizontal, axis) and is being “rotated” accordingly so that the angle between **P**B and **P**Y is kept constant.

The “biasogram” plot contains the following elements, each of which are projected into the plane using **P**: 1) The *B* vector drawn as a line from the origin, which by definition points directly along the positive vertical axis with unit length. 2) The *Y* vector drawn as a line from the origin, which by definition has unit length. In the infinitesimally likely case *Y* and *B* are completely uncorrelated, *Y* would point along the horizontal axis. 3) The individual genes *x_i•_*, which could be displayed as points for small *n*, or more typically displayed as a color-coded bivariate histogram in order to reveal the overall distribution. Finally, in lieu of skewed gridlines, we add circles centered at the origin so that the magnitude of the correlation can be estimated. Examples of the plot are presented in the figures and are discussed below.

This plot reveals several aspects of the data: First, by the angle between *Y* and *B*, we can quickly see whether the technical bias is correlated with outcome. If the angle is close to orthogonal, the bias is uncorrelated with outcome and is unlikely to increase the false positive rate. Second, from the shape of the gene cloud, we can see how strongly the genes as a group are correlated with bias and with outcome. If a substantial number of genes fall along the *B* vector, we might interpret this to indicate that the technical bias is likely to be influencing the results. Finally, from shape of the cloud around the *Y* vector we can estimate how many (if any) genes are correlated with outcome without falling into the bias cloud. Because the *B* vector points straight up, the eye can easily distinguish breaking of the left-right symmetry that would suggest that some genes may have higher-than-random correlation with outcome.

R functions to generate the biasogram plot are provided in the “Biasogram” package, available from our website at http://cbs.dtu.dk/biotools/biasogram/. The R code used to generate the figures in the case studies is available as Supporting Document S1.

## Results

### Case study 1: Docetaxel response in breast cancer

In a study published in 2003, pre-treatment biopsies were collected from 26 patients with locally advanced breast cancer, who were subsequently given neoadjuvant docetaxel [6]. Based on fraction of residual disease after treatment, 11 of these patients were defined as sensitive and 13 as resistant. Specimens were gene expression profiled on Affymetrix HG-U95Av2 microarrays, and variance filtering and *t* tests were used to identify 92 potentially discriminatory genes [6].

**Figure 2:**
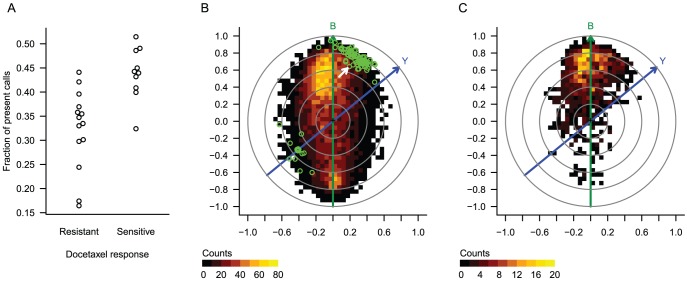
Case study 1: 26 breast tumors treated with neoadjuvant docetaxel. A) Tumor response is correlated with the fraction of present calls. B) Biasogram with a heatmap representing all 12,625 probe sets, and the 92 putatively discriminatory genes indicated in green. C) Biasogram displaying only the 1191 probe sets with variance above 2. In the biasograms, *Y* indicates the outcome vector (tumor response), *B* indicates the bias vector (fraction of present calls), the colorgram indicates a bivariate histogram of expression values, and the green circles indicate probe sets identified in the original study. The arrow indicates CYBA, the only gene that was validated in a second patient cohort.

We noticed a moderate correlation between docetaxel resistance category and the fraction of “present” calls (FPC) in the associated array (**Fig. 2A**). We have previously observed in many Affymetrix data sets that the FPC tends to be more correlated with individual gene expression levels than expected by chance, and that this correlation is expression level-dependent, suggesting that the FPC reflects a source of technical bias ([3]and data not shown). Therefore, we considered the possibility that some or all of the 92 genes may have been identified in this study as a result of confounding with technical bias, rather than a genuine biological association with drug sensitivity.

To assess the potential for confounding bias, we obtained the original dChip-normalized expression values from GEO. We defined the bias vector *B* as the FPC for each patient, and the outcome vector *Y* with *y_j_* elements as 1 or 0 if patient *j* was sensitive or resistant, respectively. We used *B* and *Y* to define a projection matrix and generated a biasogram (**Fig. 2B**). From the biasogram we first see that the *B* and *Y* vectors form a fairly acute angle, reflecting their moderate correlation. Furthermore, it is apparent that the genes generally lie along the *B* vector rather than the *Y* vector, suggesting (but not proving) that the overall gene expression profile is more influenced by technical bias than by the clinical response of interest. Finally, by inspecting the 92 discriminating genes indicated in green, we see that many of these genes are more correlated with bias than with outcome, suggesting that their identification may have been spurious. Indeed, only one of these genes, CYBA, was successfully validated in an independent cohort of 72 patients [7]. However, with an uncorrected P-value of 0.035 in the validation cohort, we consider it likely that even CYBA is not generally associated with docetaxel response, and that all of the 92 genes were originally identified only due to confounding with technical bias.

Because the authors of the original study filtered out genes with low expression or low variance before identifying discriminatory genes, we applied a similar filter and generated a second biasogram (**Fig. 2C**). We found that the shape of the data cloud was fairly similar, indicating that variance filtering did not suffice to eliminate the biased probes.

### Case study 2: T/FAC response in breast cancer

Our second example comes from a clinical trial in which 133 breast cancer patients were given neoadjuvant paclitaxel, fluorouracil, doxorubicin, and cyclophosphamide (T/FAC) chemotherapy [8]. The authors applied diagonal linear discriminant analysis (DLDA) to a training subset of 82 patients to identify a 30-gene predictor of pathological complete response (pCR) to T/FAC.

**Figure 3:**
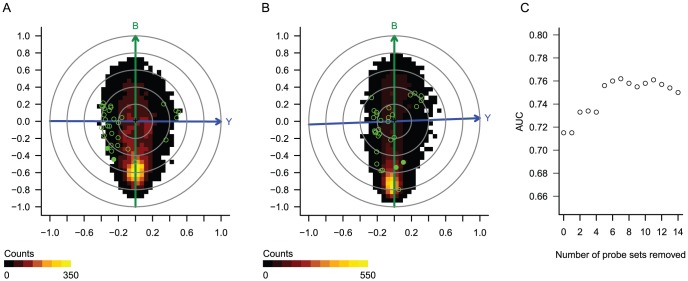
Case study 2: Breast tumors treated with neoadjuvant paclitaxel, fluorouracil, doxorubicin, and cyclophosphamide. A) Biasogram representing the training cohort of 133 tumors. B) Biasogram representing the independent validation cohort of 100 tumors. In both biasograms, *Y* indicates the outcome vector (tumor response), *B* indicates the bias vector (fraction of present calls), and the colorgram indicates a bivariate histogram of 22,283 expression values. The green open or filled circles indicate probe sets in the DLDA30 signature, with the filled circles indicating probes that, when omitted, improved the predictor's performance. C) Chart indicating area under the curve (AUC) in the validation cohort for predictors derived with the indicated number of bias-correlated probe sets omitted.

We obtained dChip-normalized expression values from the authors' webpage (http://bioinformatics.mdanderson.org/pubdata.html) and plotted a biasogram, using pCR as the outcome vector *Y* and FPC as the bias vector *B* (**Fig. 3A**) for the entire 133-patient cohort. As in the first case study, we observed many probes correlated with the bias vector *B*, confirming that technical bias has a major impact on this gene expression data set. However, in contrast to the first case study, the *B* vector lies nearly orthogonal to the outcome vector *Y*, suggesting that this type of bias is unlikely to be a confounding factor. Thus, it is not surprising that the authors' 30 genes tend to fall along the *Y* vector rather than *B* vector – and this suggests that the 30 genes are indeed “driven” by their association with clinical outcome and not by technical bias. Consistent with this hypothesis, the DLDA30 signature was confirmed in the testing cohort in the original study, as well as in an independent cohort [9].

Given that several of the DLDA30 genes show higher correlation with the bias vector than with the outcome vector, we hypothesized that it might be possible to improve the predictor by eliminating biased genes. Starting with the genes with highest absolute correlation with bias in the training cohort, we removed one gene at a time from the DLDA30 signature. For each resulting subset of the original 30 genes, we derived a new DLDA classifier and tested its performance in the independent testing cohort (Fig. 3C). We observed a small improvement in prediction performance, with the AUC increasing from 0.72 to a maximum of 0.76 when seven genes were removed, which was not a statistically significant improvement (*P* = 0.18). We explored this aspect further, using the independent validation dataset to plot a biasogram with the DLDA30 signature probes (Fig. 3B). We found that the two probes resulting in the greatest increase in DLDA signature performance, “214124_x_at” and “219741_x_at”, were correlated with the bias vector *B* in the training as well in the validation set. However, as expected, correlation with the outcome vector *Y* was high only in the training set.

### Case study 3: Platinum-based chemotherapy response in ovarian cancer

In a controversial study published in 2007, gene expression profiles from 83 advanced stage serous ovarian tumors were used to derive a predictor of response to platinum-based chemotherapy [10]. A correspondence published shortly afterwards pointed out several critical flaws [2]. The flaws were initially denied, but the article was ultimately retracted in 2012. One of the flaws described in the correspondence is that response and survival are confounded with clearly separated batches based on run date. However, the effect of this confounding on the resulting predictor was never established.

**Figure 4:**
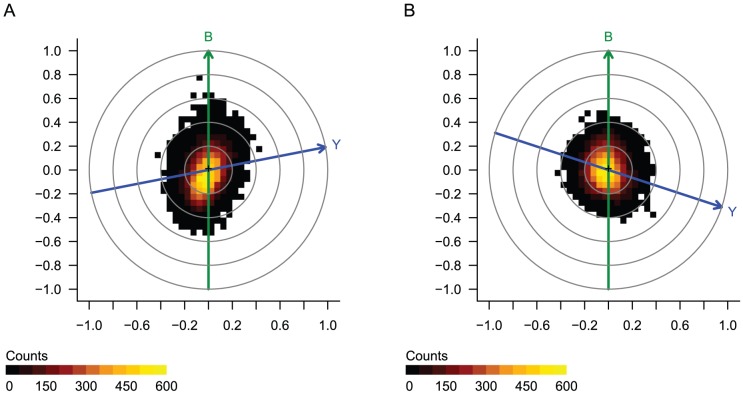
Case study 3: Ovarian tumors treated with platinum-based chemotherapy. Biasograms represent 119 tumors, with *Y* indicating the outcome vector (tumor response), and *B* indicating the bias vector defined as a binary indicator of microarray batch, as A) 2002-09-20 vs. all other dates, or B) 2002-10-23 vs. all other dates.

We obtained RMA-normalized data and clinical covariates deposited by authors from the website http://bioinformatics.mdanderson.org/Supplements/ReproRsch-Ovary/and confirmed confounding between clinical response and run date, with lowest P-values for the first two run dates, 2002-09-20 and 2002-10-23, confirming the published observations [2] (not shown). A single biasogram cannot represent a categorical bias vector with more than two levels, as is the case here. Therefore, we created biasograms in which the bias vector was an indicator variable (1 if scanned on the given date; 0 if not) for each of the two dates (Fig. 4). As expected, the Y and B vectors were visibly skewed in both. On the first run date, the bias vector seemed to have a relatively large correlation with a large number of probe sets (Fig. 4A), but this did not seem to be the case on the second run date (Fig. 4B). Therefore, we can conclude that the run date is indeed a potential confounding factor.

We then attempted to assess whether the selection of predictor genes was driven by confounding technical bias, as we did in Case Study 1. However, when we tried to indicate the original Dressmann signature probe sets, we found that they were located apparently randomly on the biasograms, with no apparent preference along either Y or B vector (not shown for clarity). Indeed, this confirms a separate flaw that was pointed out by Baggerly et al.–the lack of association between the published probe sets and clinical response. This somewhat unusual situation in which the genes in the predictor are not associated with response, most likely due to human error, prevented us from determining whether the selection of genes was driven by confounding technical bias..

## Discussion

We have described the biasogram, a method to visualize the possibly confounding relationship between a variable of interest (here, clinical outcome) and a nuisance variable (here, technical bias) on massively parallel measurements (here, gene expression profiles). The resulting plot provides a fairly intuitive overview of a large multivariate data set in which the focus is placed on how each measurement (gene) is correlated with the two variables.

The primary intended use of this method is to flag problematic data sets in which technical bias “drives” results which would otherwise be indistinguishable from genuine biological associations. We have designed and described this method in the context of gene expression microarray studies, but it this approach is potentially relevant to other matrix-type data, such as that resulting from CGH arrays, RNA-seq, etc.

In principle, this method could also be used to refine existing gene signatures by eliminating genes correlated with technical bias. We tested this idea in our Case Study 2, and observed a small improvement in predictor performance on the independent cohort. However, this improvement was not statistically significant. Without additional evidence from similarly paired data sets, we cannot give a strong recommendation for this approach.

We feel obliged to point out that correlation does not demonstrate causation: just because a gene is correlated with a technical bias does not mean that it cannot be genuinely associated with clinical outcome. However, given the large number of questionable gene expression signatures described in the literature [11], we hope that our method can help identify data sets and results that have an increased likelihood of being misleading.

One noticeable feature of the biasogram is data reduction by projecting the data matrix onto a plane; this approach is often applied in principal component analysis (PCA) and related methods for factorizing matrix data into biologically relevant components [12], [13]. Furthermore, with the addition of arrows, the biasogram is visually similar to the “biplot” commonly used in PCA [14]. The biplot is a method for plotting both rows and columns of a data matrix, as points and arrows respectively, using a projection defined by PCA. In contrast, the biasogram indicates the rows of the data matrix as points (or as a heatmap as shown here), and external variables as arrows, and the projection is defined by the external variables.

The biasogram in its current form has some limitations. First, it is necessary to identify a likely source of technical bias and define an appropriate bias vector; this may be readily apparent in some measurement systems but elusive in others. Another possible limitation is that this method is based on the assumption of linear relationships between gene expression, outcome, and bias. In reality, none of these relationships are likely to be entirely linear in nature; thus nonlinear methods for data filtering and visualization may be more sensitive to some relationships [15], [16].

## Supporting Information

Document S1 **A “Sweave” PDF file documenting the R code used to generate the figures.**
(PDF)
