# Supplementary material for: Biasogram: Visualization of Confounding Technical Bias in Gene Expression Data
Source: PLoS One. 2013 Apr 16;8(4):e61872. doi: 10.1371/journal.pone.0061872 (PMC3628873; doi:10.1371/journal.pone.0061872)
Supplement: Document S1 — A “Sweave” PDF file documenting the R code used to generate the figures. (PDF) [file pone.0061872.s001.pdf]

Supporting Document S1 for "Biasogram: visualization of confounding technical bias in gene expression data"

Marcin Krzystanek, Zoltan Szallasi and Aron C. Eklund

February 8, 2013

This "Sweave" document provides a record of the R procedures used to generate the figures in the manuscript.

1 Load and prepare packages and data

1.1 Confirm that the workspace is empty

```
> ls()
```

```
character(0)
```

1.2 Load necessary packages

```
> library(Biasogram)
> library(GEOquery)
> library(affy)
> library(beeswarm)
> library(ROC)
> library(ClassComparison)
> library(pROC)
```

Packages used can be found on CRAN or Bioconductor, except:
Class Comparison - http://bioinformatics.mdanderson.org/Software/OOMPA/
Biasogram - http://www.cbs.dtu.dk/biotools/biasogram/

1.3 Create necessary functions

Function for labelling the panels.

```
> label.panel <- function (txt, xoff = 1, yoff = xoff, cex = 8/6, font = 2)
+ {
+     x <- grconvertX(0, from = "nfc") + (xoff * strwidth("M"))
+     y <- grconvertY(1, from = "nfc") - (yoff * strheight("M"))
+     text(x, y, labels = txt, font = font, xpd = TRUE, cex = cex,
+         adj = c(0, 1))
+ }
```

Little function for checking the performance of the signature and the predictor.

```
> check.perf <- function(x)
+ {
+         tmp <- as.data.frame(x)
```

```
+         TN <- tmp[1, "Freq"] / sum(tmp[, "Freq"])*100
+         TP <- tmp[4, "Freq"] / sum(tmp[, "Freq"])*100
+         FN <- tmp[3, "Freq"] / sum(tmp[, "Freq"])*100
+         FP <- tmp[2, "Freq"] / sum(tmp[, "Freq"])*100
+         sens <- TP / (TP+FN) * 100
+         spec <- TN / (TN+FP) * 100
+         PPV  <- TP / (TP+FP) * 100
+         NPV  <- TN / (TN+FN) * 100
+         MCC  <- (TP*TN - FP*FN) / (sqrt((TP+FN)*(TN+FP)*(TP+FP)*(TN+FN)))
+         return(round(data.frame(TN,TP,FN,FP,sens,spec,PPV,NPV,MCC),2))
+ }
```

The DLDA classifier function as used in the Hess 2006 paper is found on the following web page: http://bioinformatics.mdanderson.org/Supplements/Datasets/PredictorValidation/dlda30.zip Here we extract the relevant function "myfct.dlda.short":

```
> source(pipe('sed -n 32,74p ~/Desktop/biasogram_data/dlda30/DLDA30.S'))
```

1.4 Prepare data for Figure 2

Load dchip-normalized docetaxel response dataset, directly from GEO:

```
> #gds <- getGEO('GSE6434')
> #gse6434.dchip <- gds[[1]]
> #save(gse6434.dchip, file='~/Desktop/biasogram_data/gse6434.dchip.RData')
```

The above process can take a couple minutes, so I work locally instead.

```
> (load('~/Desktop/biasogram_data/gse6434.dchip.RData'))

[1] "gse6434.dchip"
```

Load bias metrics generated in advance using "bias" package:

```
> (load('~/Data/sbge_cancer/data/misc/qc2/gse6434.qc2.RData'))

[1] "gse6434.qc2"
```

Load docetaxel signature to be plotted in Figure 1b.

```
> docetaxelSig <- c("1624_at", "36626_at", "35807_at", "38765_at", "38211_at",
+         "33133_at", "607_s_at", "31431_at", "39182_at", "32331_at", "40064_at",
+         "34305_at", "35733_at", "38784_g_at", "40619_at", "35844_at",
+         "40060_r_at", "38372_at", "39185_at", "40096_at", "40463_at",
+         "41198_at", "41627_at", "37361_at", "38618_at", "39076_s_at",
+         "33371_s_at", "1641_s_at", "36811_at", "33931_at", "38791_at",
+         "38850_at", "1635_at", "31638_at", "39347_at", "32523_at", "36846_s_at",
+         "39030_at", "691_g_at", "37674_at", "38613_at", "33781_s_at",
+         "32843_s_at", "33214_at", "922_at", "36125_s_at", "39180_at",
+         "1751_g_at", "38831_f_at", "41528_at", "40514_at", "33393_at",
+         "36208_at", "646_s_at", "1199_at", "40465_at", "39724_s_at",
+         "41757_at", "35626_at", "39561_at", "38686_at", "41551_at", "34845_at",
+         "41858_at", "35695_at", "2085_s_at", "37313_at", "38998_g_at",
+         "39812_at", "41413_at", "1997_s_at", "41308_at", "34163_g_at",
+         "39018_at", "34816_at", "41672_at", "33285_i_at", "543_g_at",
+         "40535_i_at", "41338_at", "36991_at", "32099_at", "39638_at",
+         "1008_f_at", "40888_f_at", "1250_at", "36898_r_at", "40118_at",
+         "38259_at", "38942_r_at")
```

1.5 Prepare data for Figure 3

Figure 3 was made using dchip-normalized data that can be downloaded as raw cel files from:
http://bioinformatics.mdanderson.org/Supplements/Datasets/PredictorValidation/MDA133-CELFiles.zip. Like before the process takes a few minutes so here we load previously saved data. We normalized data using "expresso" function from package "affy" using following arguments: normalize.method = "invariantset", bg.correct = FALSE, pmcorrect.method = "pmonly", summary.method = "liwong" in order to obtain expression set with dchip-like values. We also load the precomputed bias metrics.

```
> (load('~/Desktop/biasogram_data/hess.dchip.RData'))

[1] "hess.dchip"

> (load('~/Desktop/biasogram_data/hess.dchip.qc2.RData'))

[1] "hess.dchip.qc2"
```

MDACC validation dataset is available from GEO database with accession number GSE20194. We used 100 samples labelled as validation samples. Once again we use a pre-computed dchip-normalized data and bias metrics.

```
> load('~/Desktop/biasogram_data/mdaccval.dchip.RData')
> load('~/Desktop/biasogram_data/mdaccval.dchip.qc2.RData')
```

Load DLDA30 signature to be plotted in Figure 3.

```
> DLDA30 <- c("203929_s_at", "203930_s_at", "212745_s_at", "203928_x_at", "212207_at",
+          "217542_at",   "206401_s_at", "215304_at",   "219741_x_at", "204916_at",
+          "208945_s_at", "213134_x_at", "219197_s_at", "204825_at",   "205548_s_at",
+          "202204_s_at", "209617_s_at", "205354_at",   "204509_at",  "214124_x_at",
+          "213234_at",   "219051_x_at", "219044_at",   "203693_s_at", "214053_at",
+          "215616_s_at", "209773_s_at", "219438_at",   "205696_s_at", "201508_at")
```

1.6 Prepare data for Figure 4

In order to make create figure 4 we downloaded publicly available data from:
http://bioinformatics.mdanderson.org/Supplements/ReproRsch-Ovary/
This data is a supplement to Baggerly et al. 2007 paper.

```
> (load('~/Desktop/biasogram_data/ovcaRMAFromCEL.Rda'))

[1] "ovcaRMAFromCEL"

> (load('~/Desktop/biasogram_data/clinicalInfo.Rda'))

[1] "clinicalInfo"

> (load('~/Desktop/biasogram_data/celRunDate.Rda'))

[1] "celRunDate"

> (load('~/Desktop/biasogram_data/reportedGenesGEO.Rda'))

[1] "reportedGenesGEO"
```

2 Figure 1

```
> data(docMeta)
> par(lab = c(10, 10, 7), las = 1, bty = "l", xpd = NA, cex=1, mar=c(4.1, 4.1, 1.1, 2.1))
> x1 <- log2(exprs(gse6434.dchip))
> x2 <- x1["38765_at", , drop = F]
> # Outcome vector (1 - sensitive, 0 - resistant):
> y1 <- as.numeric(docMeta$response == "sensitive")
> b1 <- docMeta$present.calls
> pm1 <- getProjection(y1, b1, align = "y")
> biasogram(x = x2, y = y1, b = b1, pm = pm1, cex=1, key=F)
```

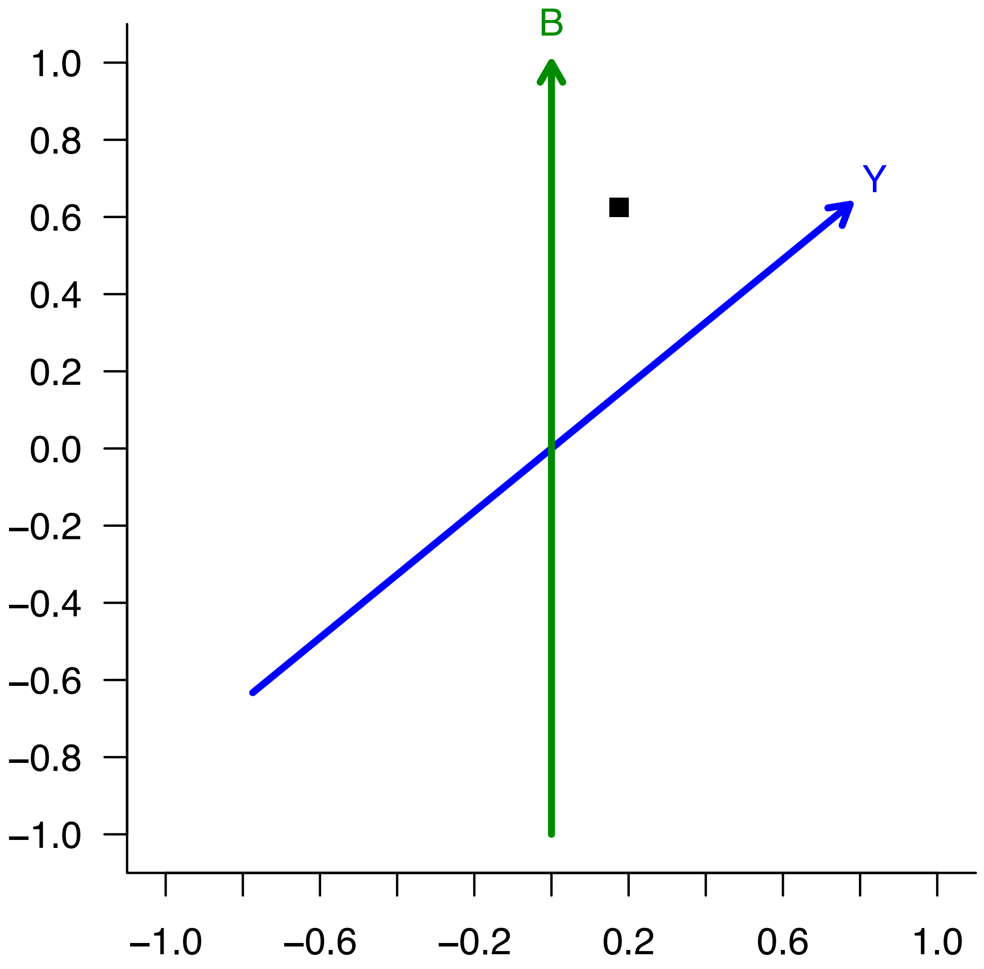

Fig. 1 was processed further in Adobe Illustrator.

3 Figure 2

```
> data(docMeta)
> layout(matrix(c(1, 2, 3), 1, 3, byrow = TRUE), widths=c(1.5, 2, 2))
> par(lab = c(10, 10, 7), las = 1, bty = "l", xpd = NA, cex=1, mar=c(5.8, 4.1, 2.8, 2.1))
> ### Fig 2a:
> beeswarm(docMeta$present.calls ~ docMeta$response == "sensitive",xlab="Docetaxel response",
+                  ylab="Fraction of present calls", labels=c("Resistant","Sensitive"))
> label.panel(txt='A')
> ### Fig 2b:
> par(lab = c(10, 10, 7), las = 1, bty = "l", xpd = NA, cex=1, mar=c(4.1, 4.1, 1.1, 2.1))
> x2 <- log2(exprs(gse6434.dchip))
> # Outcome vector (1 - sensitive, 0 - resistant):
> y2 <- as.numeric(docMeta$response == "sensitive")
> b2 <- docMeta$present.calls
> pm2 <- getProjection(y2, b2, align = "y")
> biasogram(x = x2, y = y2, b = b2, pm = pm2, cex=1, xaxt = 'n', yaxt = 'n')
> axis(1, at = seq(-1, 1, by = 0.2), labels = TRUE)
> axis(2, at = seq(-1, 1, by = 0.2), labels = TRUE)
> bullseye()
> points(project(x2[docetaxelSig, ], pm2), col = 'green')
> # CYBA gene - the only predictive gene in other independent study
> cyba <- project(x2["38765_at", ], pm2)
> adj <- 0.02
> arrows(cyba$x - 0.1 - adj,
+                  cyba$y - 0.1 - adj,
+                  cyba$x - adj,
+                  cyba$y - adj,
+                  col = 'white',
+                  length = 0.1,
+                  lwd = 2)
> label.panel(txt = 'B')
> ### Fig 2c:
> ### Only probes with variance greater than 2 are plotted.
> v2 <- apply(x2, 1, var)   #var of log2 transformed (dchip normalized data)
> x2.v <- x2[v2 > 2, ]
> dim(x2.v)

[1] 1191   24

> biasogram(x = x2.v, y = y2, b = b2, pm = pm2, cex=1)
> bullseye()
> label.panel(txt='C')
```

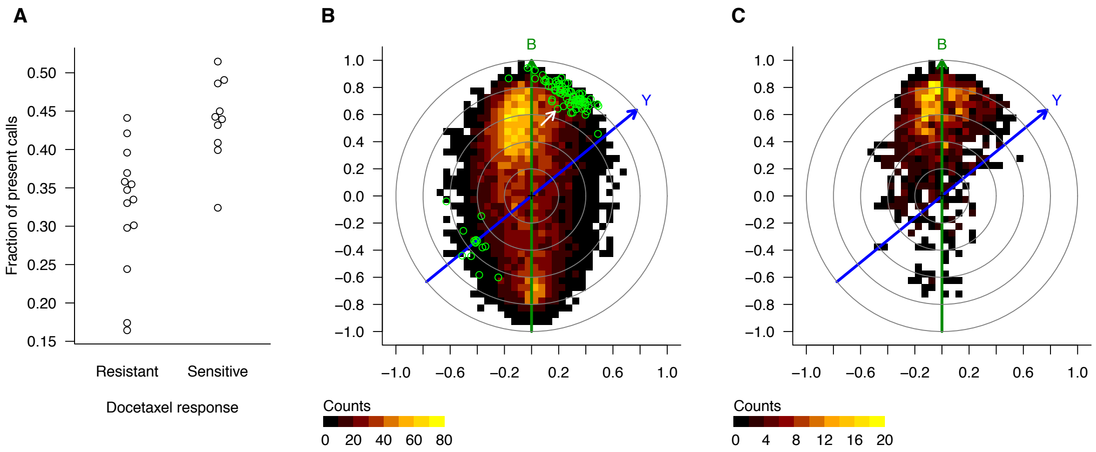
A
B
C
Fraction of present calls
0.50
0.45
0.40
0.35
0.30
0.25
0.20
0.15
Resistant
Sensitive
Docetaxel response
1.0
0.8
0.6
0.4
0.2
0.0
−0.2
−0.4
−0.6
−0.8
−1.0
−1.0
−0.6
−0.2
0.2
0.6
1.0
B
Y
Counts
0
20
40
60
80
Counts
0
4
8
12
16
20

Figure 2.

4 Figure 3

```
> layout(matrix(c(1, 2, 3), 1, 3, byrow = TRUE), widths=c(2, 2, 1.5))
> ### Fig 3a:
> x3a <- exprs(hess.dchip)
> y3a <- hess.dchip$pCR
> b3a <- hess.dchip.qc2$present.calls
> pm3a <- getProjection(y3a, b3a, align = "y")
> par(lab = c(10, 10, 7), las = 1, bty = "l", xpd = NA, cex=1, mar=c(4.1, 4.1, 1.1, 2.1))
> biasogram(x = x3a, y = y3a, b = b3a, pm = pm3a, cex=1, xaxt = 'n', yaxt = 'n')
> axis(1, at = seq(-1, 1, by = 0.2), labels = TRUE)
> axis(2, at = seq(-1, 1, by = 0.2), labels = TRUE)
> bullseye()
> #points(project(x3a[DLDA30, ], pm3a), col = 'green')
> label.panel(txt='A')
> ### The original DLDA30 signature with DLDA classifier:
> ### Figuring out biased probes:
> proj.sig <- project( x3a[DLDA30, ], pm3a)
> proj.sig.ord <- proj.sig[order(abs(proj.sig$y)), ]
> ### Now we sequentially remove the biased probes beginning from the most biased:
> class.train <- hess.dchip$pCR
> class.test <- mdaccval.dchip$pCR.vs.RD=="pCR"
> sorted.signature <- rownames(proj.sig.ord)
> current.signature <- rownames(proj.sig.ord)
> perf.table <- c()
> nr.genes.to.remove <- 15
> data.train <- exprs(hess.dchip)[sorted.signature, ]
> data.test <- exprs(mdaccval.dchip)[sorted.signature, ]
> ### Using original DLDA 30 predictor:
> original.30.genes.prediction <- myfct.dlda.short(data.train, class.train, data.test)
> ### Remove probes and compare performance:
> while (length(current.signature) > (30 - nr.genes.to.remove)){
+         data.train <- exprs(hess.dchip)[current.signature, ]
+         data.test <- exprs(mdaccval.dchip)[current.signature, ]
+         prediction <- myfct.dlda.short(data.train, class.train, data.test)
+         prediction.tbl <- table(prediction$PredictedClass, class.test)
+         perf <- check.perf(prediction.tbl)
+         perf$Removed <- 30 - length(current.signature)
+         perf$Pr.removed <- sorted.signature[length(current.signature)+1]
+         compare.roc <- roc.test(response = class.test,
+                                 predictor1 = as.vector(original.30.genes.prediction$Pro
+                                 predictor2 = as.vector(prediction$ProbOfClass1))
+         perf$AUC <- round(compare.roc$estimate[2], 3)
+         perf$p.val <- round(compare.roc$p.value, 3)
+         perf.table <- rbind(perf.table,perf)
+         current.signature <- current.signature[-length(current.signature)]
+ }
> ### Indicating points that were removed from DLDA30 signature in biasogram of the test set:
> impr <- c('214124_x_at', '219741_x_at')
> points(project(x3a[sorted.signature, ], pm3a), col = 'green')
> points(project(x3a[impr, ], pm3a), col = 'green', pch=20)
> ### Fig 3b:
> x3b <- exprs(mdaccval.dchip)
> y3b <- mdaccval.dchip$pCR.vs.RD=="pCR"
```

```
> b3b <- mdaccval.dchip.qc2$present.calls
> pm3b <- getProjection(y3b, b3b, align = "y")
> par(lab = c(10, 10, 7), las = 1, bty = "l", xpd = NA, cex=1, mar=c(4.1, 4.1, 1.1, 2.1))
> biasogram(x = x3b, y = y3b, b = b3b, pm = pm3b, cex=1, xaxt = 'n', yaxt = 'n')
> axis(1, at = seq(-1, 1, by = 0.2), labels = TRUE)
> axis(2, at = seq(-1, 1, by = 0.2), labels = TRUE)
> bullseye()
> label.panel(txt='B')
> ### Indicating points that were removed from DLDA30 signature in biasogram of the test set:
> points(project(x3b[sorted.signature, ], pm3b), col = 'green')
> points(project(x3b[impr, ], pm3b), col = 'green', pch=20)
> ### Fig 3c:
> par(lab = c(10, 10, 7), las = 1, bty = "l", xpd = NA, cex=1, mar=c(5.8, 4.1, 2.8, 2.1))
> plot(x = perf.table$Removed,
+         y = perf.table$AUC,
+         ylim = c(0.65, 0.8),
+         xlab = "Number of probe sets removed",
+         ylab = "AUC",
+         xaxt = 'n')
> axis(1, at = seq(0, 14, by = 2), labels = TRUE)
> axis(1, at =12, labels = TRUE)
> label.panel(txt='C')
```

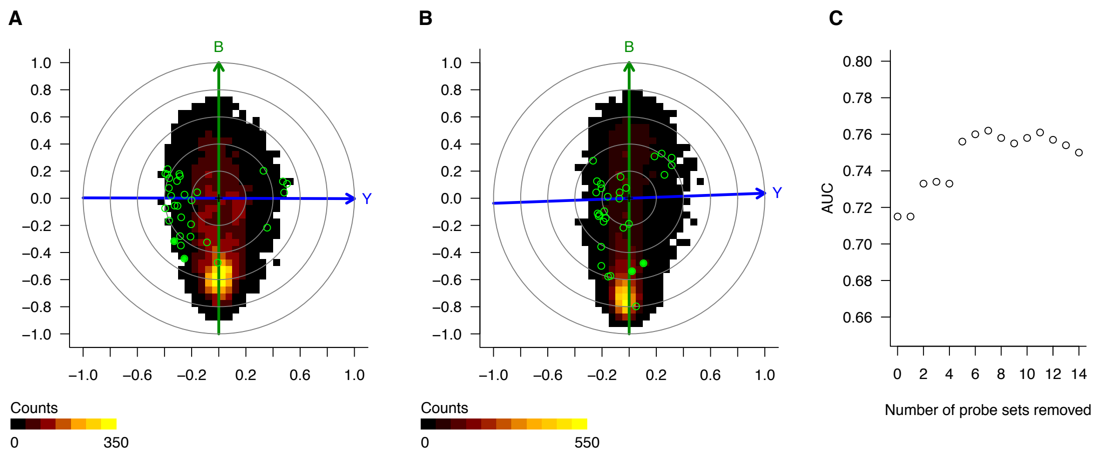

Figure 3

```
> perf.table

   TN TP FN FP  sens  spec   PPV   NPV  MCC Removed   Pr.removed   AUC p.val
1  83  1 14  2  6.67 97.65 33.33 85.57 0.09       0         <NA> 0.715 1.000
2  83  1 14  2  6.67 97.65 33.33 85.57 0.09       1    204509_at 0.715 1.000
3  83  2 13  2 13.33 97.65 50.00 86.46 0.20       2  214124_x_at 0.733 0.245
4  82  2 13  3 13.33 96.47 40.00 86.32 0.16       3    219044_at 0.734 0.183
5  82  2 13  3 13.33 96.47 40.00 86.32 0.16       4    215304_at 0.733 0.223
6  80  2 13  5 13.33 94.12 28.57 86.02 0.10       5  219741_x_at 0.756 0.304
7  79  2 13  6 13.33 92.94 25.00 85.87 0.08       6  209617_s_at 0.760 0.267
8  78  2 13  7 13.33 91.76 22.22 85.71 0.06       7  215616_s_at 0.762 0.271
9  80  2 13  5 13.33 94.12 28.57 86.02 0.10       8  203693_s_at 0.758 0.306
10 80  2 13  5 13.33 94.12 28.57 86.02 0.10       9  219051_x_at 0.755 0.346
11 81  2 13  4 13.33 95.29 33.33 86.17 0.13      10  209773_s_at 0.758 0.284
12 80  2 13  5 13.33 94.12 28.57 86.02 0.10      11  208945_s_at 0.761 0.279
```

13	80	2	13	5	13.33	94.12	28.57	86.02	0.10		12	203928_x_at	0.757	0.325
14	80	2	13	5	13.33	94.12	28.57	86.02	0.10		13	205696_s_at	0.754	0.350
15	77	2	13	8	13.33	90.59	20.00	85.56	0.05		14	205354_at	0.750	0.408

Performance table with additional measures.

5 Figure 4

```
> layout(matrix(c(1, 2), 1, 2, byrow = TRUE), widths=c(2, 2))
> par(las = 1, bty = "l", xpd = NA, cex=1, mar=c(5.8, 4.1, 2.8, 2.1))
> eset <- ovcaRMAFromCEL[, rownames(clinicalInfo)]
> celRunDate.factor <- as.factor(celRunDate)
> ### Fig 4a
> i <- "2002-09-20"
> y <- (clinicalInfo$Response == "CR")
> b <- ifelse(celRunDate.factor == i, 2, 1)
> x <- eset
> proj <- biasogram(x = x, y = y, b = b, cex = 1, xaxt = 'n', yaxt = 'n')
> axis(1, at = seq(-1, 1, by = 0.2), labels = TRUE)
> axis(2, at = seq(-1, 1, by = 0.2), labels = TRUE)
> bullseye(seq(0.2, 1, by = 0.2))
> label.panel(txt='A')
> ### Fig 4b
> i <- "2002-10-23"
> y <- (clinicalInfo$Response == "CR")
> b <- ifelse(celRunDate.factor == i, 2, 1)
> x <- eset
> proj <- biasogram(x = x, y = y, b = b, cex = 1, xaxt = 'n', yaxt = 'n')
> axis(1, at = seq(-1, 1, by = 0.2), labels = TRUE)
> axis(2, at = seq(-1, 1, by = 0.2), labels = TRUE)
> bullseye(seq(0.2, 1, by = 0.2))
> label.panel(txt='B')
```

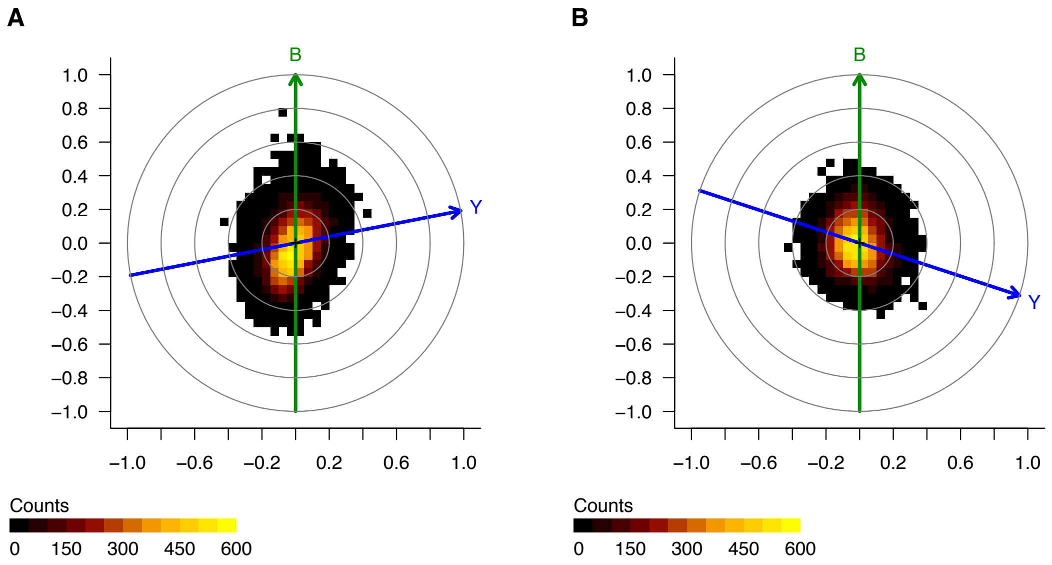

6 System information

6.1 R sessionInfo

The results in this file were generated using the following packages:

```
> sessionInfo()

R version 2.15.0 (2012-03-30)
Platform: x86_64-apple-darwin9.8.0/x86_64 (64-bit)

locale:
[1] C/UTF-8/C/C/C/C

attached base packages:
[1] splines   stats     graphics  grDevices utils     datasets  methods
[8] base

other attached packages:
 [1] pROC_1.5.4            plyr_1.7.1            ClassComparison_2.15.0
 [4] PreProcess_2.12.2     oompaBase_2.15.0      ROC_1.32.0
 [7] beeswarm_0.1.1        affy_1.34.0           GEOquery_2.23.5
[10] Biobase_2.16.0        BiocGenerics_0.2.0    Biasogram_0.1.0
[13] squash_1.0.5

loaded via a namespace (and not attached):
[1] BiocInstaller_1.4.9   RCurl_1.91-1          XML_3.9-4
[4] affyio_1.24.0         preprocessCore_1.18.0 tools_2.15.0
[7] zlibbioc_1.2.0
```

6.2 Other information

```
> system('uname -v', intern = TRUE)

[1] "Darwin Kernel Version 10.8.0: Tue Jun  7 16:33:36 PDT 2011; root:xnu-1504.15.3~1/RELEASE_I38

> system('pdftex --version', intern = TRUE)[1]

[1] "pdfTeX 3.1415926-2.3-1.40.12 (TeX Live 2011)"
```
